# Total calcium-sensing receptor expression in circulating monocytes is increased in rheumatoid arthritis patients with severe coronary artery calcification

**DOI:** 10.1186/s13075-014-0412-5

**Published:** 2014-08-19

**Authors:** Julien Paccou, Cédric Boudot, Cédric Renard, Sophie Liabeuf, Said Kamel, Patrice Fardellone, Ziad Massy, Michel Brazier, Romuald Mentaverri

**Affiliations:** Department of Rheumatology, Amiens University Hospital, Place Victor Pauchet, FR-80054 Amiens, France; INSERM U1088 ‘Pathophysiological mechanisms and consequences of cardiovascular calcification: role of cardiovascular and bone remodelling’, Avenue René Laennec, Amiens University Hospital, FR-80054 Amiens, France; Research Clinic Centre, Amiens University Hospital, Place Victor Pauchet, FR-80054 Amiens, France; Department(s) of Nephrology, Amboise Paré University Hospital, 9 Avenue Charles de Gaulle, FR-92100 Boulogne Billancourt, France; Department of Endocrine and Bone Biology, Amiens University Hospital, Avenue René Laennec, FR-80054 Amiens, France

## Abstract

**Introduction:**

Human circulating monocytes express the calcium-sensing receptor (CaSR) and are involved in atherosclerosis. This study investigated the potential association between vascular calcification in rheumatoid arthritis (RA) and CaSR expression in circulating monocytes.

**Methods:**

In this cross-sectional study, 50 RA patients were compared to 25 control subjects matched for age and gender. Isolation of peripheral blood mononuclear cells and flow cytometry analysis were performed to study the surface and total CaSR expression in circulating monocytes. Coronary artery calcium (CAC) and abdominal aortic calcification (AAC) scores were evaluated by computed tomography and an association between these scores and the surface and/or total CaSR expression in circulating monocytes in RA patients was investigated.

**Results:**

The two groups were similar in terms of age (RA: 60.9 ± 8.3 years, versus controls: 59.6 ± 5.3 years) and gender (RA: 74.0% females versus 72.0% females). We did not find a higher prevalence and greater burden of CAC or AAC in RA patients versus age- and gender-matched controls. When compared with control subjects, RA patients did not exhibit greater total CaSR (101.6% ± 28.8 vs. 99.9% ± 22.0) or surface CaSR (104.6% ± 20.4 vs. 99.9% ± 13.7) expression, but total CaSR expression in circulating monocytes was significantly higher in RA patients with severe CAC (Agatston score ≥200, n = 11) than in patients with mild-to-moderate CAC (1 to 199, n = 21) (*P* = 0.01).

**Conclusions:**

This study demonstrates for the first time that total CaSR expression in human circulating monocytes is increased in RA patients with severe coronary artery calcification.

## Introduction

Patients with rheumatoid arthritis (RA) are exposed to a substantially increased risk of cardiovascular events (especially coronary heart disease) and death [1–5]. Furthermore, the increased risk for cardiovascular disease cannot be fully explained by traditional cardiovascular risk factors [1,2]. Moreover, stratification tools such as the Framingham risk score [6], widely used in primary prevention in the general population, are less suitable in RA patients [7,8]. A growing body of evidence suggests that nontraditional risk factors such as chronic inflammation and RA features have a pivotal role in accelerated atherosclerosis and the increased cardiovascular disease risk [9–11].

Vascular calcification is commonly used as a subclinical marker of atherosclerosis and has been linked to increased all-cause mortality, cardiovascular mortality and coronary events [12]. Moreover, patients with RA are known to develop early-onset, widespread calcification in various vascular beds [13–15]. This is consistent with the concept whereby inflammation promotes atherosclerosis and vascular calcification [16]. Cardiovascular disease remains a major problem in RA patients and there is a need to focus on further delineating the underlying biological mechanisms involved in vascular calcification, and developing and evaluating novel biomarkers.

Patients with chronic kidney disease (CKD) are also at a particularly high risk for cardiovascular disease. The prevalence and severity of vascular calcification is correlated with disease stage in CKD and considered as a cardiovascular risk marker. Vascular calcification is the result of both passive and active processes that involve a variety of factors and proteins [17]. Originally cloned from bovine parathyroid glands, the calcium-sensing receptor (CaSR) has been studied for its role in mediating systemic calcium homeostasis. However, the CaSR has also been shown to have pleiotropic actions on cells, including modification of cellular proliferation, differentiation, and apoptosis [18–20]. Interestingly, the CaSR was expressed on vascular smooth muscle cells (VSMCs) and its expression was decreased in the arteries of patients with CKD compared to controls [21]. Moreover, modulation of CaSR expression on VSMCs in CKD by calcimimetic agents (R-568) was demonstrated to effectively delay progression of vascular calcification and atherosclerosis in uremic apolipoprotein E-deficient mice [22].

However, in patients with RA, the role of CaSR expression remains to be investigated. Monocytes/macrophages are known to play a crucial role in the pathogenesis of atherosclerosis and a considerable focus has been placed on their precursor, circulating monocytes as predictors of cardiovascular disease [21–23]. Intriguingly, CaSR expression has been demonstrated in human circulating monocytes and extracellular calcium has been shown to be a chemokinetic agent for human circulating monocytes [24–26]. Moreover, monocytes/macrophages also modulate vascular calcification *in vitro* [27]. It seemed important to clarify the role of the CaSR expressed in circulating monocytes in the pathological process leading to vascular calcification in patients with RA.

In a previous study, we provided evidence indicating that CaSR expression can be measured by flow cytometry in human circulating monocytes and its measurement would be potentially useful in certain clinical situations, in which changes in CaSR expression could be expected [28]. The present study was therefore designed to explore the potential association between vascular calcification in RA and CaSR expression in human circulating monocytes.

## Methods

### Study design

In this cross-sectional study, 50 RA patients were compared to 25 non-RA control subjects matched for age and gender. Control subjects were enrolled from a pool of volunteers set up by the general Clinical Center of Amiens University Hospital, France. RA patients were enrolled from the Rheumatology Outpatients Department of Amiens University Hospital, France. All subjects gave their written informed consent, and the study was approved by the University Hospital ethics committee (*Comité de Protection des Personnes Nord-Ouest 2*) (Number 2012-A00323-40) and *l’Agence Française de Sécurité Sanitaire des Produits de Santé*.

### Study population

Inclusion criteria for this study were as follows: presence of the 2010 American College of Rheumatology/European League Against Rheumatism classification criteria for RA, age 45 to 80 years and capacity to understand the goals of the study. Only anti-TNFα was allowed as current and/or previous biological disease-modifying anti-rheumatic drugs (bDMARDs). Exclusion criteria were as follows: self-reported or physician-diagnosed history of myocardial infarction, heart failure, coronary artery revascularization, stroke events, peripheral vascular disease, abdominal aortic aneurysm and current atrial fibrillation, weight exceeding 150 kg, inflammatory diseases other than RA. Controls did not meet the classification criteria for RA or any other inflammatory disease.

### Study protocol

Information was obtained by means of a structured interview, physical examination, laboratory tests, and electron-beam computed tomography (CT), and the medical records of all patients were reviewed. Demographic and clinical characteristics and traditional cardiovascular risk factors were collected. The general cardiovascular risk profile was estimated by the Framingham risk scores (FRS) [6]. In RA patients, duration of disease was recorded and disease activity was measured using the disease activity score based on evaluation of 28 joints (DAS28). The DAS28 is a validated composite index containing a 28-joint count for tenderness (TJC), a 28-joint count for swelling (SJC), erythrocyte sedimentation rate (ESR) or high-sensitivity C-reactive protein (hs-CRP), and the patient’s overall assessment of well-being (range 0 to 100). DAS28 was adjusted on hs-CRP. The health assessment questionnaire (HAQ) was also filled in. Current and previous biological and conventional synthetic DMARDs used for RA were determined both from information provided by the patients and from medical records. Concomitant medications, such as NSAIDs and corticosteroids, were also recorded.

### Laboratory parameters

RA patients and controls were required to fast overnight prior to collection of blood for determination of complete blood cell count as well as measurement of serum calcium (Ca), phosphate (Ph), protein (Pt), creatinine (Scr) and glycemia. Serum intact parathyroid hormone (iPTH 1-84) was determined by a chemiluminometric immunoassay (Liaison N-tact PTH CLIA; Diasorin, Stillwater, MN, USA). Serum 25 OH vitamin D level was determined (LIAISON™ 25 OH Vitamin D Total Assay; Diasorin). Corrected calcium levels were calculated for each subject. Estimated glomerular filtration rate (GFR) was calculated using the modification of diet in renal disease (MDRD) formula (ml/min). Total cholesterol, high-density lipoprotein (HDL) cholesterol, low-density lipoprotein (LDL) cholesterol and triglycerides were determined. The Westergren ESR was used and hs-CRP values were analyzed in an onsite biochemistry laboratory using standard autoanalyzer techniques. The presence of rheumatoid factor (RF) as well as anti-cyclic citrullinated peptide antibody (anti-CCP) was only determined for RA patients. IL6 and TNFα were assayed (eBiosciences™ hIL6 total enzyme-linked immunosorbent assay (ELISA) BMS213/2 kit and hTNFα total ELISA BMS2034 kit; Vienna, Austria). As indicates in the manufacturer’s kit instruction, the limits of detection of hTNFα and hIL6 are 5.0 pg/ml and 0.92 pg/ml.

### Traditional cardiovascular risk factors

Subjects were considered to present hypertension when they were taking antihypertensive agents or when they had a systolic blood pressure ≥140 mmHg and/or diastolic pressure ≥90 mmHg. Blood pressure was determined as the average of two measurements obtained at an interval of five minutes after subjects had been resting in the supine position for at least 10 minutes. Diabetes mellitus was defined as the presence of any of the following: (i) fasting blood glucose ≥1.26 g/l (7 mmol/l), (ii) current use of oral hypoglycemic agents and/or use of insulin. Smoking status was recorded as self-reported current smokers. Height and weight were measured and body mass index (BMI) was calculated as weight/height^2^ (kg/m^2^). A family history of coronary artery disease was defined as a first-degree relative with myocardial infarction before the age of 55 years in men, and before the age of 65 years in women. Patients and controls were considered to have hypercholesterolemia when they self-reported a previous diagnosis of hypercholesterolemia, and/or use of lipid-lowering drugs.

### Study outcomes

This study evaluated (i) coronary artery calcification (CAC) and abdominal aortic calcification (AAC) scores and (ii) surface and total CaSR expression in human circulating monocytes.

#### CAC and AAC scores

In order to quantify the presence and extent of vascular calcification, each patient underwent a nonenhanced 64-slice CT scan (Discovery CT750 HD; GE Healthcare, Milwaukee, WI, USA). A prospective electrocardiogram (ECG)-gated sequential scan of the whole heart was performed to measure CAC with the following parameters: collimation of 64 × 0.625 mm, slice thickness of 2.5 mm, gantry rotation time of 350 ms, tube voltage of 120 kV and a tube current of 200 mA. CAC scores were calculated according to the Agatston method, using CAC-scoring software (Smart Score 4.0; GE Healthcare). CAC scores were defined as none (CAC = 0) mild-to-moderate (CAC = 1 to 199) or severe (CAC ≥200). The limit of CAC severity score was set at 200 because a calcium score ≥200 among patients ≥50 years old provided strong evidence that patients of either sex had obstructive coronary artery disease (CAD) [29]. Moreover, the mean CAC score for RA patients was almost 200. Abdominal aorta was evaluated without ECG gating. The start of volume acquisition was placed at the level of the diaphragmatic aortic hiatus and the end was placed below the aortic bifurcation. Scanning parameters were: collimation of 64 × 0.625 mm, slice thickness of 0.625 mm, a pitch of 1, gantry rotation time of 500 ms, tube voltage of 120 kV and tube current of 300 mA. Image sets for all patients were analyzed with a commercially available external workstation (Advantage Windows 4.6; GE Healthcare). The abdominal aorta was segmented manually on three-dimensional MIP images. In order to reduce errors due to noise, a cutoff of 160 HU was applied. The total calcification volume was calculated as the sum of all voxels in the remaining volume. The abdominal aorta calcification CT scan score was calculated as follows: ((total calcification volume)/(aortic wall surface area) × 100)) [30].

#### Surface and total CaSR expression in human circulating monocytes

Isolation of peripheral blood mononuclear cells was performed for flow cytometry analysis. For each participant, 3 ml of blood was mixed with 22 ml of PBS-0.5%BSA (mononuclear cells were isolated by density gradient centrifugation); 25 ml of diluted blood were carefully added to 10 ml of Lymphosep™, and the tubes were then centrifuged for 25 minutes at room temperature and 1800 g. After centrifugation, the interphase containing mononuclear cells was carefully aspirated and the cells were washed using 5 ml of PBS-0.5%BSA before being centrifuged for five minutes at room temperature (1800 g). Cells were finally mixed with 1.8 ml of fetal bovine serum (FBS) and 0.2 ml of dimethyl sulfoxide (DMSO) before being aliquoted into four 0.5 ml tubes and frozen at −80°C. For the flow cytometry analysis, after washes in PBS-0.5%BSA, cells were incubated with anti-Calcium Sensing Receptor Monoclonal Antibody (5C10, ADD; Thermo Fisher Scientific, Rockford, IL, USA) or with negative control Mouse IgG2a (X 0943; DakoCytomation, Glostrup, Denmark) for 30 minutes on ice. After washes in PBS-0.5%BSA, cells were incubated with PE-conjugated IgG mouse antibodies (Polyclonal Goat Anti-Mouse Immunoglobulins/RPE (R 0480; DakoCytomation)) for 30 minutes on ice in the dark. After washes in PBS-0.5%BSA, cells were incubated with monoclonal CD14 antibodies, human, conjugated to FITC (130-080-701, MACS; Miltenyi Biotec, Bergisch Gladbach, Germany) or monoclonal Mouse IgG2a isotype control antibodies conjugated to FITC (130-091-837, MACS; Miltenyi Biotec) for 30 minutes on ice. After several washes, surface and total CaSR expressions were analyzed by FACSAria cytometer (BD Biosciences). When assessing total CaSR expression, detection of intracellular antigens requires a cell permeabilization step prior to immunostaining. When necessary, cells were therefore incubated with 100 μl of BD Cytofix/Cytoperm™ (Cat. No. 554722) solution for 20 min on ice and were then washed twice with BD Perm/Wash buffer (Cat. No. 554723), before being exposed to both primary and secondary antibodies and assessed for total CaSR expression. Both total and surface CaSR expression, that is fluorescence intensity, are presented as a percentage. Rheumatoid arthritis patients were compared to control subjects who served as reference (fluorescence intensity is equal to 100% in control subjects).

### Statistical analysis

The sample size was estimated using the nonparametric method developed by Noether [31]. To demonstrate that the probability of RA patients with CAC (≥1) has a total and/or surface CaSR expression greater than RA patients without CAC (=0) is at least 0.73 with alpha = 5% and beta = 20%, 25 RA patients with and 25 RA patients without CAC are necessary. This probability of 0.73 corresponds approximately to a Cohen’s effect size of 0.86 [32]. Quantitative variables are expressed as mean ± standard deviation (SD) or median [range] and qualitative variables are expressed as percentage. Student’s *t* test (or Wilcoxon test as appropriate) was used for comparison of quantitative variables between the two groups. Chi-square or Fisher’s exact test was used for comparison of qualitative variables between the two groups. A multivariate linear regression model was used for assessment of independent variables correlated with both surface and total CaSR expression. The correlation between CaSR expression and laboratory parameters (Ca, Ph, creatinine clearance, hsCRP, 25 OH vitamin D, iPTH, total cholesterol, LDL cholesterol, HDL cholesterol, HIL6, and HTNFα) was studied with the Spearman’s rank correlation and a correlation was considered only when the coefficient was between 0.41 and 1.00 or between −0.41 and −1.00, with a significant *P* value. Two-sided *P* values <0.05 were considered significant in multivariate analysis. SAS software version 9.2 (SAS Institute, Cary, NC, USA) software was used for all analyses.

## Results

### Clinical and demographic characteristics

The clinical and demographic characteristics of the patients with RA and the control subjects are shown in Tables 1 and 2. All patients were of Caucasian descent. The RA and control groups did not differ significantly in terms of age *(*RA: 60.9 ± 8.3; controls: 59.6 ± 5.3*)*, gender or the prevalence of traditional cardiovascular risk factors, but the prevalence of diabetes mellitus was twofold higher among control subjects (12% versus 6%). Moreover, the mean duration of disease was greater than 12 years, more than one half of RA patients were treated by TNFα antagonists. Remission or low disease activity (defined as a DAS28-hsCRP ≤3.2) was observed in 40 (80.0%) patients. Compared to control subjects, RA patients did not exhibit higher total CaSR (101.6% ± 28.8 vs. 99.9% ± 22.0) or surface CaSR (104.6% ± 20.4 vs. 99.9% ± 13.7) expression in human circulating monocytes. Mean ESR and mean serum urea were significantly higher in RA patients than in control subjects (*P* = 0.001 and *P* = 0.006, respectively), while monocyte count was higher in RA patients than in control subjects (*P* = 0.005) (Table 2). HIL6 levels were not detectable for 25 control subjects and 45 RA patients and ranged between 0.97 and 86.8 pg/ml for five RA patients. HTNFα levels were not detectable for 22 control subjects and 34 RA patients and were increased in three control subjects (up to 110.1 pg/ml) and 16 RA patients (up to 351.0 pg/ml).

**Table 1:** **Demographic and clinical characteristics of study population**

**Characteristics**	**RA patients (n = 50)**	**Control subjects (n = 25)**	***P***
Age, years	60.9 ± 8.3	59.6 ± 5.3	0.42
Gender (female), %	74.0%	72.0%	0.85
Body mass index, kg/m^2^	27.0 ± 6.0	28.9 ± 8.3	0.32
Current smokers, %	16.0%	16.0%	1.00
Diabetes, %	6.0%	12.0%	0.36
Family history of cardiovascular disease, %	18.0%	20.0%	0.83
Hypertension, %	64.0%	48.0%	0.50
Current use of antihypertensive therapy, %	44.0%	36.0%	0.50
Systolic blood pressure, mmHg	126.2 ± 17.0	129.3 ± 18.4	0.45
Diastolic blood pressure, mmHg	81.0 ± 12.3	82.6 ± 11.6	0.59
Hypercholesterolemia, %	36.0%	48.0%	0.74
Current use of statins, %	24.0%	16.0%	0.32
Current use of fibrates, %	2.0%	16.0%	**0.02**
FRS, %	11.4%	13.8%	0.36
RF and/or CCP antibodies, %	86.0%	-	
Erosive arthritis, %	54.0%	-	
Duration of disease (years)	12.2 ± 8.5	-	
HAQ	0.8 ± 0.7	-	
DAS28-hs-CRP	2.5 ± 0.9	-	
DAS28-hs-CRP ≤3.2, %	80.0%	-	
Current methotrexate users, %	90.0%	-	
Current anti-TNFα users, %	58.0%	-	
Current NSAIDs users, %	30.0%	-	
Current corticosteroid users, %	26.0%	-	

Values are the mean ± standard deviation (SD) (significant results are indicated in bold). RA, rheumatoid arthritis; FRS, Framingham risk score; RF, rheumatoid factor; CCP, citrullinated cyclic protein; HAQ, health assessment questionnaire; DAS28-hs-CRP, disease activity score 28 high-sensitivity C-reactive protein; TNFα, tumor necrosis factor alpha; NSAIDs, nonsteroidal anti-inflammatory drugs.

**Table 2:** **Biochemical characteristics of the study population**

**Characteristics**	**RA patients (n = 50)**	**Control subjects (n = 25)**	***P***
Hemoglobin, g/dl	13.7 ± 1.5	14.3 ± 1.1	0.12
White blood cell, mm^3^	6264 ± 1797	5624 ± 1650	0.13
Neutrophil, mm^3^	3604 ± 1458	3232 ± 1283	0.26
Monocytes, mm^3^	440 ± 145	352 ± 112	**0.005**
Urea, mmol/l	6.1 ± 1.8	5.2 ± 1.0	**0.006**
Creatinine, μmol/l	64.6 ± 14.0	66.7 ± 10.6	0.46
Creatinine clearance, ml/mn	97.6 ± 21.0	92.8 ± 15.5	0.26
Calcium, mmol/l	2.31 ± 0.07	2.31 ± 0.09	0.81
Phosphorus, mmol/l	0.94 ± 0.17	0.98 ± 0.15	0.32
Glycemia, mmol/l	4.27 ± 1.08	4.94 ± 0.92	**0.01**
hs-CRP, mg/liter	3.9 ± 5.2	3.1 ± 4.8	0.54
ESR rate, mm/hour	12.1 ± 10.0	6.3 ± 4.2	**0.001**
25 OH vitamin D, ng/ml	17.8 ± 8.4	19.9 ± 8.8	0.30
iPTH, pg/ml	43.6 ± 25.0	42.2 ± 18.1	0.41
Total cholesterol, g/l	2.13 ± 0.36	2.29 ± 0.37	0.51
LDL cholesterol, g/l	1.26 ± 0.29	1.36 ± 0.27	0.18
HDL cholesterol, g/l	0.65 ± 0.15	0.61 ± 0.19	0.42
Triglycerides, g/l	1.10 ± 0.51	1.33 ± 0.58	0.09

Values are the mean ± standard deviation (SD) (significant results are indicated in bold). hs-CRP, high-sensitivity C-reactive protein; ESR, erythrocyte sedimentation rate; iPTH, intact parathyroid hormone; LDL cholesterol, low-density lipoprotein; HDL cholesterol, high-density lipoprotein.

### Vascular calcification

The prevalence of vascular calcification and the CAC and AAC scores in patients with RA and control subjects are shown in Table 3. Coronary artery calcification was not more prevalent in patients with RA (64.0%) than in controls (52.0%) (*P* = 0.31). The mean CAC score was 205 ± 443 in patients with RA and 134 ± 248 in controls (*P* = 0.29). Abdominal aortic calcification was not more prevalent in patients with RA (66.6%) than in controls (64.0%) (*P* = 0.96). The mean AAC score was 1.8 ± 2.0 in patients with RA and 1.7 ± 1.6 in controls (*P* = 0.73).

**Table 3:** **Prevalence of vascular calcification and mean calcification scores in patients with RA and in controls**

**Site**	**Prevalence of vascular calcification**			**Mean ± SD calcification score**		
	**RA**	**Controls**	***P*** **value**	**RA**	**Controls**	***P*** **value**
**Coronary**	64.0%	52.0%	0.31	205 ± 443	134 ± 248	0.29
**Aorta**	66.0%	64.0%	0.96	1.8 ± 2.0	1.7 ± 1.6	0.73

RA, rheumatoid arthritis; SD, standard deviation.

### Linear regression analysis and correlations

Spearman’s rank correlation failed to distinguish any significant correlation as prespecified between surface and total CaSR expression and laboratory parameters in RA patients. Indeed, no association was found between age, gender or clinical parameters with both surface and total CaSR expression.

### CaSR expression and vascular calcification

Correlations with log-transformed data for CAC and AAC scores and total and surface CaSR expression were investigated. While no significant correlation was found between log-transformed data for CAC score and total CaSR expression, these data were close to significance (*P* = 0.07). No other significant correlations were observed between log-transformed data for CAC score and surface CaSR expression (*P* = 0.87) or between log-transformed data for AAC score and total and surface CaSR expression (*P* = 0.49 and *P* = 0.67 respectively).

Speculating that total and surface CaSR expression in human circulating monocytes could be different between RA patients with mild-to-moderate or severe coronary artery calcification (CAC = 1 to 199 versus CAC ≥200 or CAC = 30 to 199 versus CAC ≥200 with a limit of detection for CAC set at 30 instead of 0), subgroup analysis was also performed. Total CaSR expression in human circulating monocytes was significantly higher in RA patients with severe CAC than in patients with mild-to-moderate CAC, whether the limit of detection was set at 0 (*P* = 0.01) or 30 (*P* = 0.03) (Table 4). No significant differences were observed when CAC was compared to surface expression of CaSR on monocytes. A multivariate logistic regression model was used for assessment of independent variables associated with mild-to-moderate or severe CAC. Only male gender was associated with severe CAC in the multivariate model with odds ratio = 0.034 (95% CI: 0.003 to 0.391; *P* = 0.007) (Table 5).

**Table 4:** **CAC scores and CaSR expression in RA patients**

	**CAC 1-199 (n = 21)**	**CAC ≥200 (n = 11)**	
**Total CaSR expression, %**	91.7 ± 20.7	115.3 ± 31.8	**0.01**
	**CAC 1–199**	**CAC ≥200**	
	**(n = 21)**	**(n = 11)**	
**Surface CaSR expression, %**	104.2 ± 15.0	100.7 ± 8.5	0.41
	**CAC 30–199**	**CAC ≥200**	
	**(n = 12)**	**(n = 11)**	
**Total CaSR expression, %**	91.0 ± 19.4	115.2 ± 31.8	**0.03**
	**CAC 30–199**	**CAC ≥200**	
	**(n = 12)**	**(n = 11)**	
**Surface CaSR expression, %**	101.8 ± 13.9	100.7 ± 8.5	0.83

Values are the mean ± standard deviation (SD) (significant results are indicated in bold). CAC, coronary artery calcium; CaSR, calcium-sensing receptor; RA, rheumatoid arthritis.

**Table 5:** **Logistic regression analysis of mild-to-moderate CAC versus severe CAC**

	***P*** **value for univariate model**	***P*** **value for multivariate model**
Age	0.59	
Gender	**0.003**	**0.007**
Weight	0.66	
Height	**0.048**	0.91
Body mass index	0.48	
Hypertension	1.00	
Hypercholesterolemia	0.29	
Diabetes	1.00	
Current smoker	1.00	
Methotrexate users	1.00	
Anti-TNFα users	1.00	
NSAIDs users	0.37	
Corticosteroid users	0.21	
Erosive arthritis	1.00	
RF and/or CCP antibodies	1.00	
hs-CRP	0.32	
TNFα	0.66	
DAS28-hs-CRP	**0.01**	0.73
Systolic blood pressure, mmHg	0.87	
Diastolic blood pressure, mmHg	0.12	0.42
Serum calcium	0.85	
Serum phosphate	0.09	
GFR-MDRD	**0.03**	0.08
Glycemia	0.71	
Total cholesterol, g/l	0.84	
LDL cholesterol, g/l	0.77	
HDL cholesterol, g/l	0.74	
Serum intact PTH	0.33	
Serum 25 OH Vitamin D	0.15	0.43
Hemoglobin	0.31	
White blood cell	0.57	
Neutrophils	0.69	
Monocytes	**0.04**	0.11
Surface CaSR expression	0.84	
Total CaSR expression	**0.03**	0.90

Significant results are indicated in bold. CaSR, calcium-sensing receptor; hs-CRP, high-sensitivity C-reactive protein; PTH, parathyroid hormone; RF, rheumatoid factor; CCP, citrullinated cyclic protein; DAS28-hs-CRP, disease activity score 28-high-sensitivity C-reactive protein; TNFα, tumor necrosis factor alpha; NSAIDs, nonsteroidal anti-inflammatory drugs; GFR-MDRD, estimated glomerular filtration rate using the modification of diet in renal disease; LDL cholesterol, low-density lipoprotein; HDL cholesterol, high-density lipoprotein.

## Discussion

Calcium-sensing receptor is expressed fairly ubiquitously and is known to regulate numerous cellular processes ranging from fertilization to cell death [18–20]. Calcium-sensing receptor should therefore not only be associated with the G-protein coupled receptor responsible for calcium homeostasis in complex organisms. Moreover, clear evidence has been published that vitamin D3 concentrations, inflammatory cytokines and particularly secondary hyperparathyroidism modify CaSR expression throughout the body [33]. However, apart from *in vitro* and *in vivo* experiments, no technique is available to provide clinicians with an assessment of ‘CaSR status’ in patients. In this context, we hypothesized that CaSR expression in human circulating monocytes could represent an accessible biomarker in the clinic [28].

As previously demonstrated, CaSR expression is influenced by the direct microenvironment of the cells and particularly by phosphate, calcium, vitamin D3, inflammatory cytokines and chemokines [33]. It can be speculated that CaSR expression in CD14+ monocytes may, to a certain degree, reflect the influence of circulating blood on CaSR expression in other cell types in physiological and pathological conditions. Confirming our previous results, this study assessed the CaSR status of patients by flow cytometry in monocytes isolated from total blood. Moreover, total CaSR expression in human circulating monocytes was increased in RA patients with severe CAC versus patients with mild-to-moderate CAC. These results contrast with those obtained by Malecki *et al*. [34], who demonstrated that CaSR expression on the surface of circulating monocytes isolated from atherosclerotic patients was significantly decreased compared to atherosclerotic and type 2 diabetic patients as well as control subjects. The twofold higher prevalence of diabetic mellitus and the significant difference in terms of fasting glucose concentrations between control subjects and RA patients may therefore constitute a possible confounder for interpretation of CaSR protein levels. However, taken together, these studies indicate that CaSR expression is modified during atherosclerotic processes and that CaSR status could be a particular valuable marker to be studied on isolated monocytic cells. A similar discrepancy was observed between our study and that performed in atherosclerotic or atherosclerotic and type 2 diabetic patients in terms of the percentage of CD14+ cells, which were shown to express surface CaSR. However, direct comparison of these studies remains difficult, as different anti-CaSR antibodies were used, therefore corresponding to different immunoreactivities. Further studies are needed to clarify this point.

We did not find a higher prevalence and greater burden of CAC or AAC in RA patients versus age- and gender-matched controls. Probably due to the limited sample size of this study and to the higher-than-expected prevalence and burden of CAC or AAC in control subjects, these effects were not statistically significant. It should be noted that the control subjects of our study were older than those reported in other studies comparing control subjects versus RA patients [13,14]. Moreover, the majority of RA patients were in clinical remission or presented low disease activity with a very low degree of inflammation evaluated by ESR, hs-CRP, IL6 and TNFα. ESR, IL6 and TNFα concentrations have been demonstrated to be significantly associated with higher coronary calcium levels in RA [10,14]. To our knowledge, this is the first study to investigate AAC in RA patients and our results suggest the absence of any significant difference in AAC score between RA patients and controls.

In view of the calcium concentration present in vascular calcification, it was important to clarify whether CaSR plays a role in this pathological process. Recently, considerable focus has been placed on circulating cells, as circulating monocytes, involved in cardiovascular disease [23] and CaSR expression has been demonstrated in circulating monocytes [25,26]. We have previously shown that CaSR expression in circulating monocytes could represent an accessible biomarker to monitor an individual’s ‘CaSR status’ under nonpathological conditions [28]. Confirming our previous observations, the present study demonstrates, for the first time, a relationship between total CaSR expression in circulating monocytes and the severity of CAC in RA. Although this finding needs to be confirmed in other pathological conditions in which CaSR expression is known to be modulated - that is during secondary hyperparathyroidism in CKD patients - our data demonstrate that the CaSR status of patients can be monitored by following CaSR expression in circulating monocytes over time. It is important to note that, endogenous agonists of CaSR, such as Mg2+, spermine and cyclic amino acids, may also modify the net plasma membrane concentration of the receptor *in vivo*, without reaching 5 mM Ca2+ [35]. Initially described in HEK cells overexpressing CaSR and referred to as the ADIS phenomenon [36], this process could be of physiological or pathophysiological relevance, as recently demonstrated by Hénaut *et al*. [37]. However, the net increase in plasma membrane CaSR predominantly results from an increase in anterograde trafficking via the secretory pathway - at a constant rate of endocytosis - and the proportion of CaSR expressed at the plasma membrane may differ significantly from cell to cell. Further clinical studies must be conducted to more clearly define the physiological role of CaSR in circulating monocytes. Ionic calcium has been shown to exert a chemotactic effect on human monocytes [27]. Whether or not the calcium contained in areas of vascular calcification are involved in the homing of circulating monocytes via CaSR stimulation remains to be demonstrated. It can nevertheless be assumed that the calcium gradient adjacent to the vascular wall would be correlated with the degree of vascular calcification, which would explain why total CaSR expression is increased in RA patients with severe CAC scores. Noteworthy, CaSR is also expressed by VSMCs and CaSR expression was decreased in the arteries of patients with CKD compared to controls [21]. In CKD, the prevalence of vascular calcification is also high and modulation of CaSR expression on VSMCs by calcimimetic agents (R-568) has been recently demonstrated to effectively delay progression of vascular calcification and atherosclerosis in uremic apolipoprotein E-deficient mice and secondary endpoints of the ADVANCE study [22,38].

Although this study was designed to assess a potential link between CaSR expression and vascular calcifications in RA patients, it presents a number of limitations. First, with a cross-sectional design, a temporal relationship between total CaSR expression and coronary artery calcification cannot be proven. Second, this study failed to show that the prevalence of coronary artery calcification and coronary calcium scores were higher in RA patients who were in remission for most of them. Finally, the assessment of vascular calcification by spiral computed tomography allows assessment of calcified atherosclerotic plaque, but does not evaluate the other components of atherosclerotic plaque.

## Conclusions

The potential relationship between subclinical atherosclerosis assessed by vascular calcification and total and/or surface CaSR expression in human circulating monocytes should therefore be confirmed by future studies. Moreover, our experiments only focused on human circulating monocytes. Further studies are necessary to investigate whether simultaneous modification of CaSR expression is observed in both human circulating monocytes and VSMCs during the vascular calcification process. CaSR expression in human circulating monocytes could be considered to be an emerging tool to monitor CaSR status of individuals with different diseases including vascular calcification.

## Abbreviations

AAC: abdominal aortic calcification
anti-CCP: anti-cyclic citrullinated peptide antibody
bDMARDs: biological disease-modifying anti-rheumatic drugs
BMI: body mass index
Ca: calcium
CAC: coronary artery calcium
CAD: coronary artery disease
CaSR: calcium-sensing receptor
CKD: chronic kidney disease
CT: computed tomography
DAS28: disease activity score based on evaluation of 28 joints
DMSO: dimethyl sulfoxide
ECG: electrocardiogram
ELISA: enzyme-linked immunosorbent assay
ESR: erythrocyte sedimentation rate
FBS: fetal bovine serum
FRS: Framingham risk score
GFR: glomerular filtration rate
HAQ: health assessment questionnaire
HDL: high-density lipoprotein
hs-CRP: high-sensitivity C-reactive protein
IgG: immunoglobulin G
IL: interleukin
iPTH: intact parathyroid hormone
LDL: low-density lipoprotein
MDRD: modification of diet in renal disease
NSAIDs: nonsteroidal anti-inflammatory drugs
Ph: phosphate
Pt: protein
RA: rheumatoid arthritis
RF: rheumatoid factor
Scr: creatinine
SJC: swollen joint count
TJC: tender joint count
TNFα: tumor necrosis factor alpha
VSMCs: vascular smooth muscle cells
